# The Effect of Body Mass Index on the Prevalence of Gastrointestinal Symptoms Among a Saudi Population

**DOI:** 10.7759/cureus.17751

**Published:** 2021-09-06

**Authors:** Saad Alkhowaiter, Rawan M Alotaibi, Khulood K Alwehaibi, Arwa Aljohany, Batoul Alruhaimi, Munira Almasaad, Sulaiman A Alshammari, Majid A Alsahafi

**Affiliations:** 1 Gastroenterology, College of Medicine, King Khalid University Hospital, Riyadh, SAU; 2 Gastroenterology, College of Medicine, King Saud University, Riyadh, SAU; 3 Family and Community Medicine, College of Medicine, King Saud University, Riyadh, SAU; 4 Internal Medicine, King Abdulaziz University Faculty of Medicine, Jeddah, SAU

**Keywords:** body mass index, obesity, gastrointestinal symptoms, saudi arabia, overweight

## Abstract

Background: While multiple studies have evaluated the effect of body mass index (BMI) on the prevalence of gastrointestinal (GI) symptoms, data from Saudi Arabia are scarce. This study aimed to evaluate the association between GI symptoms and BMI in a Saudi population.

Methods: A prospective cross-sectional study was conducted between September 2019 and April 2020. The data were collected using an electronic self-administered questionnaire. The study included adult participants and collected data on patients’ demographics and GI symptoms. Participants with underlying GI diseases were excluded. A multivariate regression analysis was used to report the adjusted prevalence of GI symptoms in different BMI categories.

Results: A total of 4415 participants completed the survey. After applying the exclusion criteria, 3866 were included. The mean age was 26.3 (±8.8) and 58.2% were females. The mean BMI was 25.2 (±6.7), and the distribution of BMI was as follows: underweight 428 (11.1%), normal BMI 1789 (46.2%), overweight 912 (23.5%), and obese 737 (19.1%). After adjustment for age, gender, and smoking and coffee habits, obesity (BMI > 30) was significantly associated with heartburn (*p* < 0.01, aOR 1.6, 95% CI: 1.33 - 1.92), bloating (*p* < 0.01, aOR 1.31, 95% CI: 1.08 - 1.6), and diarrhea (*p* < 0.01, aOR 1.72, 95% CI: 1.36 - 2.17)). Underweight (BMI < 18.5) was significantly associated with abdominal pain (*p* < 0.01, aOR 1.4, 95% CI: 1.12 - 1.73), nausea (*p* < 0.01, aOR 1.6, 95% CI: 1.29 - 2.1), and vomiting (*p* < 0.01, aOR 2.02, 95% CI: 1.23 - 3.25). There was no significant association between BMI and constipation.

Conclusion: Obesity was associated with heartburn, diarrhea, and bloating, while underweight status was associated with nausea, vomiting, and abdominal pain. No association between BMI and constipation was found.

## Introduction

Obesity is a major health concern worldwide, and data from the World Health Organization show that obesity has increased dramatically from 1975 to 2016 for both males and females [1]. In Saudi Arabia, about 69.6% of adults are overweight, and 34.7% are obese, which makes Saudi Arabia one of the countries with the highest prevalence of obesity in the world [2]. Moreover, the prevalence of obesity is projected to increase to 41% in men and 78% in women by 2022 [3]. Obese individuals are at higher risk of some gastrointestinal (GI) symptoms based on self-reported studies [4-6]. Previous studies have shown that an increase in body mass index (BMI) could increase the risk for upper and lower GI symptoms [4-6]. For example, the prevalence of frequent dysphagia, abdominal pain, and altered bowel habits in obese subjects was 32%, 40%, and 82%, respectively, compared to 17%, 25%, and 61% of normal-weight individuals [4]. Moreover, the frequency and severity of gastroesophageal reflux disease (GERD) symptoms are strongly associated with BMI [7,8]. In addition, multiple studies have shown a significant relationship between higher BMI and diarrhea and a negative relation with constipation [6,9-12]. However, most of these studies did not consider various environmental and cultural factors. GI symptoms in high BMI individuals have a considerable impact on the quality of the patient’s life. Furthermore, they lead to a considerable economic burden due to the frequent utilization of medical services for consultation, testing, and therapeutic interventions. While the association between BMI and GI symptoms has been well characterized in multiple international studies, local data are scarce and such associations have not been explored in a Saudi population. Given the variations in social and cultural factors, international data may not be applicable to the Saudi population. Therefore, the study aimed to evaluate the effect of BMI on the prevalence of different GI symptoms in a Saudi population.

## Materials and methods

A prospective cross-sectional study was performed between September 2019 to April 2020. The Institution Review Board at King Khalid University Hospital, Riyadh, Saudi Arabia approved the study protocol. All participants voluntarily agreed to participate in the study and provided informed consent. An invitation to complete an online self-administered survey was distributed through the social media platforms, Twitter and WhatsApp. The invitation included an embedded link to the survey. The survey was created using a Google form document and completed anonymously by the respondents.

This study included Saudi participants who were 18 years or older and who agreed to participate in the study. Participants who had the following conditions were excluded: chronic GI diseases, such as celiac and inflammatory bowel disease; prior history of intra-abdominal surgery other than appendectomy and cholecystectomy; and systemic disorders with possible GI manifestations, such as multiple sclerosis and stroke. This step was taken to avoid these condition confounding symptoms and concentrate on obesity's effect on gastrointestinal symptoms.

For the assessment of GI symptoms, a translated Arabic version of a previously validated survey was used for the same purpose. The questionnaire consisted of two parts. The first part included sociodemographic details (age, gender, region, area, education, occupation, marital status, smoking history, coffee, and alcohol intake). The second part focused on GI symptoms, which included nausea, vomiting, heartburn, diarrhea, bloating, constipation, and abdominal pain. Height and weight were recorded as reported by the participants. The participants were categorized according to their BMI: underweight (BMI < 18.5), normal weight (BMI 18.5-24.9), overweight (BMI 25-29.9), or obese (≥ 30) [13].

Statistical analysis

The percentage and count were used for categorical variables, while the mean and standard deviation were used for continuous variables as appropriate. Univariate analysis was performed using the Chi-square test and the Student t-test for categorical variables and continuous variables, respectively. Multivariate logistic regression analysis was used to examine the association between BMI categories and different GI symptoms after adjustment for gender, age, and smoking and coffee habits, which have been associated with GI symptoms in prior studies [9]. A two-tailed p-value < 0.05 was considered statistically significant. Statistical analysis was performed using the R statistical software, version 1.2.5042 - © 2009-2020 RStudio, Inc., Boston, MA.

## Results

A total of 4415 respondents agreed to participate. After excluding 544, a total of 3866 subjects were included. The mean age was 26.3 (±8.8), and 58.2% were females. The mean BMI was 25.2 (±6.7). The distribution of BMI was as follows: underweight 428 (11.1%), normal BMI 1789 (46.2%), overweight 912 (23.5%), and obese 737 (19.1%). Table 1 shows the characteristics of the participants and the distribution of different variables.

**Table 1:** Baseline characteristics n=3866

	Mean	
Age	26.3 (±8.8)	
BMI	25.2 (±6.7)	
	n	%
Gender		
Female	2251	58.2
Male	1615	41.8
Region		
Center	1853	47.9
East	488	12.6
North	243	6.3
South	312	8.1
West	970	25.1
Area		
City	3032	78.4
Governorate	637	16.5
Village	197	5.1
Marital status		
Divorced	68	1.8
Married	998	25.8
Single	2793	72.2
Widow	7	0.2
Occupation		
Employer	1144	29.6
Not employer	643	16.6
Retired	66	1.7
Self-employment	96	2.5
Student	1917	49.6
Educational level		
Postgraduate	223	5.8
Bachelor	2514	65
High school	1091	28.2
Intermediate	30	0.8
Elementary	6	0.2
Illiterate	2	0.1
Coffee intake		
Yes	1801	46.6
No	2065	53.4
Smoking		
Yes	724	18.7
No	3142	81.3
Alcohol		
Yes	130	3.4
No	3736	96.6

Among all included subjects, the prevalence of GI symptoms was as follows: abdominal pain 39.86%, nausea 17.95%, vomiting 3.1%, heartburn 37.32%, bloating 68.31%, diarrhea 16.01%, and constipation 37.58%. In univariate analysis, obesity (BMI > 30) was associated with older age (p < 0.01) and male gender (p < 0.01). Underweight status was associated with the female gender (p < 0.01). Table 2 shows the distribution of different variables in different BMI categories.

**Table 2:** Unadjusted association between body mass index and gastrointestinal symptoms *Significant ***Highly significant

	Abdominal pain			Heartburn		
	n/n %	OR	P-value	n/n %	OR	P-value
		(95% CI)			(95% CI)	
Underweight	205/428 (47.9%)	1.4134784	0.00138*	158/428 (36.92%)	1.1509612	0.209
		(1.1431390–1.7472648)			(0.9229275–1.4317659)	
Normal	705/1789 (39.41%)	Reference		603/1789 (33.71%)	Reference	
Overweight	343/912 (37.61%)	0.9268768	0.36457	351/912 (38.49%)	1.230585	0.014*
		(0.7862324–1.0918305)			(1.0425401–1.4517897)	
Obese	288/737 (39.10%)	0.9862484	0.87727	331/737 (44.91%)	1.6035014	1.26e-07***
		(0.8268650–1.1752394)			(1.3457035–1.9103712)	
	Nausea			Vomiting		
	n\n %	OR	P-value	n\n %	OR	P-value
		(95% CI)			(95% CI)	
Underweight	125/428 (29.21%)	1.7774775	2.56e-06***	26/428 (6.07%)	2.03907734	0.00355*
		(1.3957157–2.2549652)			(1.24500775–3.2581427)	
Normal	337/1789 (18.84%)	Reference		55/1789 (3.07%)	Reference	
Overweight	122/912 (13.38%)	0.6653796	0.000375***	20/912 (2.19%)	0.70688952	0.18944
		(0.5300091–0.8306554)			(0.41150348–1.1666608)	
Obese	110/737 (14.93%)	0.7558957	0.019449*	19/737 (2.58%)	0.83428716	0.50186
		(0.5957192–0.9529937)			(0.47966645–1.3897081)	
	Bloating			Diarrhea		
	n\n %	OR	P-value	n\n %	OR	P-value
		(95% CI)			(95% CI)	
Underweight	287/428 (67.06%)	1.014321	0.901	61/428 (14.25%)	0.9863232	0.929
		(0.8118514–1.271774)			(0.7244409–1.3249809)	
Normal	1194/1789 (66.74%)	Reference		258/1789 (14.42%)	Reference	
Overweight	646/912 (70.83%)	1.210218	0.031*	142/912 (15.41%)	1.0943421	0.427
		(1.0183224–1.440545)			(0.8745793–1.3647810)	
Obese	514/737 (69.74%)	1.148606	0.143	158/737 (21.44%)	1.619325	1.73e-05***
		(0.9551387–1.384077)			(1.2980387–2.0155037)	
	Constipation					
	n\n %	OR	P-value			
		(95% CI)				
Underweight	170/428 (39.72%)	1.0900444	0.434			
		(0.8773910–1.3516350)				
Normal	674/1789 (73.67%)	Reference				
Overweight	329/912 (36.07%)	0.9335602	0.416			
		(0.7907393–1.1012056)				
Obese	280/737 (37.99%)	1.0135771	0.881			
		(0.8488147–1.2090676)				

Association between BMI and GI Symptoms

Among obese individuals, the prevalence of nausea (14.93%), vomiting (2.58%), heartburn (44.91%), abdominal pain (39.1%), bloating (69.74%), diarrhea (21.44%), and constipation (37.99%) was higher compared to normal BMI subjects. For normal BMI individuals, the prevalence of GI symptoms was as follows: nausea 18.84%, vomiting 3.07%, heartburn 33.71%, abdominal pain 39.41%, bloating 66.74%, diarrhea 14.42%, and constipation 37.67%.

Table 3 shows the univariate analysis between BMI and GI symptoms. Obesity (BMI > 30) was significantly associated with heartburn (p < 0.01, OR 1.6, 95% CI: 1.35 - 1.91), nausea (p < 0.01, OR 0.76, 95% CI: 0.6 - 0.95), and diarrhea (p < 0.01, OR 1.62, 95% CI: 1.3 - 2). Overweight status (BMI 25 - 29.9) was significantly associated with heartburn (p < 0.01, OR 1.23, 95% CI: 1.04 - 1.45), nausea (p < 0.001, OR 0.67, 95% CI: 0.53 - 0.83), and bloating (p < 0.05, OR 1.21, 95% CI: 1.02 - 1.44). Underweight (BMI < 18.5) was significantly associated with abdominal pain (p < 0.01, OR 1.41, 95% CI: 1.14 - 1.75), nausea (p < 0.01, OR 1.78, 95% CI: 1.4 - 2.25), vomiting (p < 0.01, OR 2.04, 95% CI: 1.25 - 3.26). There was no significant association between BMI and constipation.

**Table 3:** Association between body mass index and gastrointestinal symptoms adjusted for age, gender, smoking, and coffee *Adjusted for age, gender, and tobacco use

	Abdominal pain	P-value*	Heartburn	P-value*	
	OR* (95% CI)		OR* (95% CI)		
Underweight	0.6898645 (1.1294907–1.7363304)	0.00212	0.4947974 (0.9343668–1.4530207)	0.171	
Normal	Reference		Reference		
Overweight	1.4005859 (0.8506070–1.2007518)	0.90178	1.1666090 (1.0274439–1.4505025)	0.0231	
Obese	1.0109108 (0.9558476–1.3875333)	0.1367	1.2211024 (1.3340731–1.9276195)	4.87E-07	
	Nausea		Vomiting		
	OR*	P-value*	OR*	P-value*	
	(95% CI)		(95% CI)		
Underweight	0.3017262 (1.2911259–2.1051443)	5.67E-05	0.03268754 (1.23098766–3.25427675)	0.004259	
Normal	Reference		Reference		
Overweight	1.6516623 (0.7019312–1.1227322)	0.32984	2.02561012 (0.45281964–1.33615043)	0.398594	
Obese	0.8898773 (0.8549096–1.4082740)	0.45361	0.79305687 (0.54591376–1.66545404)	0.923076	
						Bloating		Diarrhea		
	OR*	P-value*	OR*	P-value*	
	(95% CI)		(95% CI)		
Underweight	2.1560078 (0.7981452–1.2572690)	0.99985	0.1566206 (0.7270219–1.3327373)	0.953	
Normal	Reference		Reference		
Overweight	1.0000218 (1.0874286–1.5646276)	0.004309	0.9909002 (0.9156083–1.4501820)	0.221	
					Obese	1.3032764 (1.0824918–1.6020582)	0.006095	1.1541307 (1.3695401–2.1739560)	3.51E-06	
	Constipation	P-value*								
	OR* (95% CI)				
Underweight	0.6531714 (0.8574391–1.3284841)	0.55408			
Normal	Reference				
Overweight	1.0682819 (0.8522383–1.2070363)	0.8705			
Obese	1.0145765 (0.9813569–1.4286467)	0.0772			

After adjustment for age, gender, and smoking and coffee habits, obesity (BMI ≥ 30) was significantly associated with heartburn (p < 0.01, aOR 1.6, 95% CI: 1.33 - 1.92), bloating (p < 0.01, aOR 1.31, 95% CI: 1.08 - 1.6), and diarrhea (p < 0.01, aOR 1.72, 95% CI: 1.36 - 2.17). Overweight status (BMI 25 - 29.9) was significantly associated with heartburn (p < 0.05, aOR 1.17, 95% CI: 1.03 - 1.45) and bloating (p < 0.01, aOR 1, 95% CI: 1.09 - 1.56). Underweight status (BMI < 18.5) was significantly associated with abdominal pain (p < 0.01, aOR 1.4, 95% CI: 1.12 - 1.73), nausea (p < 0.01, aOR 1.6, 95% CI: 1.29 - 2.1), and vomiting (p < 0.01, aOR 2.02, 95% CI: 1.23 - 3.25). There was no significant association between BMI and constipation.

## Discussion

This study evaluated the association between BMI and GI symptoms. A positive relationship was found between high BMI and heartburn, diarrhea, and bloating. Additionally, low BMI was significantly associated with nausea, vomiting, and abdominal pain. A similar conclusion was reached by some but not all previous studies. This variation could be explained by many factors that can contribute to these different findings, such as population characteristics, the influence of genes, environmental interactions, and cultural habits [14].

Obese individuals are more prone to have heartburn compared to other BMI groups, which was consistent with other studies [7,8]. This could be due to the pressure difference between the abdomen and the lower esophageal sphincter. The increase in intra-abdominal pressure causes gastric acid to flow back into the esophagus [8]. Increased BMI was not associated with nausea and vomiting, which was also found in another similar study [15], while other studies reported different findings [6,9]. Nausea and vomiting were higher in the underweight group in our sample, which may require further investigation. It is conceivable that these patients lost weight due to an underlying undiagnosed disease that could be the cause of nausea and vomiting.

Being obese could increase the risk for upper and lower abdominal pain, but the effect of obesity in upper abdominal pain is more noticeable [9]. Some studies only linked obesity with upper abdominal pain and found no significant association with pain in the lower hemiabdomen [15]. Furthermore, most studies did not differentiate between upper and lower abdominal pain since many patients found it challenging to make this distinction. In those studies, there was a positive relationship between high BMI and abdominal pain [4,5]. In contrast, our study showed no significant relationship between high BMI and abdominal pain, which aligned with findings from other studies [11,16]. Interestingly, the prevalence of abdominal pain in our study was high among underweight subjects. Therefore, we hypothesized that weight loss and abdominal pain in these individuals could be resulting from an underlying undiagnosed pathology.

In line with previous studies, we found that high BMI was associated with diarrhea and bloating, while there was no association with constipation [6,9,11,12]. The pathophysiology of diarrhea in obese individuals could be attributed to several possible mechanisms such as dietary habits, which may accelerate colonic transit time [17], intestinal inflammation, and changes in bile acids resulting in bile acid diarrhea [5]. Unsurprisingly, bloating was the most reported GI symptom, and the prevalence was higher in the overweight group. However, the mechanism behind this finding was not clear. It is possible that obese people may not be able to differentiate between true bloating and their abdominal fat [18]. This raises the question if bloating is an under-diagnosed symptom in obese individuals.

Interestingly, no association between high BMI and constipation was found despite the fact that there is a well-established relationship between a sedentary lifestyle and constipation. One study conducted with a Turkish adult population associated functional constipation with high BMI [19]. Nevertheless, many studies, including ours, have found no significant association between obesity and constipation [6,9,11,12]. However, constipation might be under-reported. In one study, 54% of patients did not complain of constipation, even though they had evidence of fecal impaction on the X-ray [20]. Consequently, it could be under-recognized since it might not be as bothersome as the other symptoms. Overall, the inconsistency of outcomes might be explained by cytokines theory, where obesity is considered a chronic inflammatory condition in which several cytokines play an important role in the pathogenesis of altered GI motility [21].

The current study is the first study to evaluate the effect of BMI on the prevalence of GI symptoms in a Saudi population, and a relatively large number of subjects were included. However, there were some limitations of the study. The sampling of subjects was not random, and a relatively large proportion of younger subjects led to the under-representation of older age groups. Being online, using a self-administered questionnaire are some of the limitations. Also, the lack of control subjects is another limitation. The measurement of BMI was dependent on self-reported height and weight, which may not have optimal accuracy. Similarly, the GI symptoms were collected as reported by respondents. Furthermore, other factors could affect the prevalence of GI symptoms that we did not account for in our study, such as medications.

## Conclusions

The research was aimed to explore any associations between BMI and common GI symptoms. It had been found that the higher the BMI, the more symptoms are likely to occur, and this is highly similar to the published literature. Therefore, obesity in Saudi Arabia was associated with heartburn, diarrhea, and bloating, while underweight status was associated with nausea, vomiting, and abdominal pain. At the same time, no association between BMI and constipation was found. However, further studies are warranted to explore factors that could explain these associations.
